# The Epigenome View: An Effort towards Non-Invasive Prenatal Diagnosis

**DOI:** 10.3390/genes5020310

**Published:** 2014-04-09

**Authors:** Elisavet A. Papageorgiou, George Koumbaris, Elena Kypri, Michael Hadjidaniel, Philippos C. Patsalis

**Affiliations:** 1 NIPD Genetics Ltd., Neas Engomis 31, Engomi, Nicosia 2409, Cyprus; E-Mails: e.papageorgiou@nipd.com (E.A.P.); g.koumbaris@nipd.com (G.K.); e.kypri@nipd.com (E.K.); 2 The Cyprus Institute of Neurology and Genetics, 6 International Airport Avenue, Ayios Dometios, Nicosia 2370, Cyprus; E-Mail: michaelh@cing.ac.cy

**Keywords:** non-invasive prenatal diagnosis, epigenetic modifications, free fetal DNA, maternal circulation, chromosomal aneuploidies

## Abstract

Epigenetic modifications have proven to play a significant role in cancer development, as well as fetal development. Taking advantage of the knowledge acquired during the last decade, great interest has been shown worldwide in deciphering the fetal epigenome towards the development of methylation-based non-invasive prenatal tests (NIPT). In this review, we highlight the different approaches implemented, such as sodium bisulfite conversion, restriction enzyme digestion and methylated DNA immunoprecipitation, for the identification of differentially methylated regions (DMRs) between free fetal DNA found in maternal blood and DNA from maternal blood cells. Furthermore, we evaluate the use of selected DMRs identified towards the development of NIPT for fetal chromosomal aneuploidies. In addition, we perform a comparison analysis, evaluate the performance of each assay and provide a comprehensive discussion on the potential use of different methylation-based technologies in retrieving the fetal methylome, with the aim of further expanding the development of NIPT assays.

## 1. Introduction

The discovery of free fetal DNA in maternal circulation [1] was a landmark towards the development of non-invasive prenatal diagnostic assays, and remarkable advances have taken place since then. The revolution was initiated in 1997 with the determination of the fetal fraction, which was estimated to be 3% during the early stages of the pregnancy [2]. In the following years, more advanced technologies were used (e.g., digital PCR) to re-evaluate the fetal DNA fraction, which is now estimated to be 10%–20% [3]. 

Deciphering the critical characteristics of the fetal genome has been the main goal for the development of non-invasive prenatal tests (NIPT). Studies have shown that the origin of maternal free DNA present in maternal peripheral blood is the hematopoietic system of the mother [4]. On the other hand, free fetal DNA (ffDNA) is derived from embryonic cell degradation in maternal peripheral blood [5,6] or from apoptotic placental cells [7,8,9]. More recent studies have confirmed the above, using bisulfite sequencing technologies and provided convincing evidence for the origin of both fetal and maternal free DNA in maternal plasma [10]. It has also been demonstrated that free fetal DNA from maternal plasma is cleared immediately (within a few hours) after pregnancy [11]. These findings were confirmed by more recent studies [12,13,14,15] and is a finding of great importance, since the presence of fetal DNA from previous pregnancies would interfere with the correct interpretation of subsequent pregnancies. A number of independent studies have also demonstrated that the amount of fetal DNA released in maternal circulation increases with pregnancy progression [2,16].

Other studies characterizing ffDNA have found that the size of fetal DNA fragments were estimated to be <0.3 kb, whereas that of maternal DNA was >1 kb [17,18]. Follow-up studies have demonstrated that the release of fetal DNA is due to the apoptosis of no more than three nucleosomal complexes, and it has been shown that the average fetal fragment size is 286 ± 28 bp with a maximum ffDNA fragment size ranging from 219 to 313 bp [19]. However, better determination and characterization of free fetal DNA fragment sizes will allow further evaluation of the diagnostic limitations that are introduced because of fragment size.

The first attempts towards NIPT were based on the use of fetal-specific markers, which were easily distinguishable in maternal circulation, as they were fetal-specific. Such markers were Y-chromosome-specific loci for fetal sex determination, such as DYS14 [1,20], as well as fetal Rhesus D found in maternal circulation in pregnancies in which the mother was Rhesus D negative [21,22]. These methods were readily and rapidly introduced in the clinical setting of diagnostic laboratories worldwide [23], and within a few years, the field of NIPT evolved even further with the use of Y-chromosome-specific markers or paternally inherited polymorphic loci for the NIPT of X-linked inherited diseases, as well as through the identification of fetal-specific chromosomal translocations [24] and trinucleotide repeats in muscular dystrophy (DMPK) [25].

The above successful developments relied on the presence or absence of a fetal-specific marker. However, further developments and advances were needed for the identification of fetal specific-markers that are independent of gender and polymorphic sites and would allow direct discrimination of the free fetal DNA from the free maternal DNA [23,26]. The challenge of the field was the development of NIPT for the detection of chromosomal aneuploidies in the fetus. The need for the identification of fetal-specific markers that would enable the discrimination of a diploid pregnancy from an aneuploid pregnancy was urgent, because aneuploidies are among the most frequent fetal abnormalities, the most common of which are trisomy 21, trisomy 18, trisomy 13 and aneuploidies associated with chromosomes X and Y [23,27]. Major efforts took place from a number of independent research groups towards the NIPT of the most common chromosomal aneuploidies [23,26,28]. One such area that was extensively investigated was epigenetic modifications during development and how such changes could be taken into consideration for the identification of methylation fetal-specific markers that could potentially be used for the development of NIPT of fetal chromosomal abnormalities. In this review, we describe, compare and evaluate the different epigenetic-based approaches that have been implemented in the field of NIPT of fetal aneuploidies.

## 2. DNA Methylation in Fetal Development

DNA methylation is an enzymatic chemical modification of the genome, which includes the addition of a methyl group to the carbon-5 position of the cytokines of CpG dinucleotides [29]. 

The methylation pattern of the cell is reset during embryogenesis, and it is established early during development [30,31]. After its establishment, the methylation pattern is inherited from one cell generation to the next [29]. The methylation occurs in CpG dinucleotides non-uniformly distributed in the genome. In contrast, areas rich in CpG dinucleotides (CpG Islands) are usually found in promoter regions of genes, and the majority of them are presented as non-methylated [29]. It is estimated that the human genome consists of approximately 30,000 CpG islands, of which, a proportion of 50%–60% lies within promoters [32]. Although the majority of these sequences are non-methylated, the CpG islands of imprinted genes and the inactive X chromosome are predominantly methylated [33].

DNA methylation is a dynamic process and may change during the post-developmental stage [34]. It is believed that 60% of tissue-specific differentially methylated regions (TDMRs) are methylated in embryonic cells, while during the differentiation of embryonic tissues to adult tissues, they undergo de-methylation [35,36,37,38,39]. More recent studies confirm the above, indicating that some of the methylated TDMRs undergo de-methylation in embryonic cells during the transformation into adult tissues, while a large proportion remains methylated in newborn tissues [40]. Therefore, the de-methylation of TDMRs occurs at a later developmental stage. In addition, the results indicated that specific regions of the genome show a different methylation pattern in different tissues and at different stages of development. The above findings provided convincing evidence that fetal DNA will present different methylation patterns from the methylation pattern of the maternal DNA. 

Several independent research groups argued that methylation patterns are different between different tissues [41,42,43,44]. In 2008, a team of researchers led by Beck implemented a newly developed methodology known as MeDIP (methylated DNA immunoprecipitation), which was used in combination with whole genome microarray technologies to investigate the methylation status of all known promoter regions and CpG islands in different tissues [44]. Based on the above study, the phenomenon of CpG islands’ methylation in normal cells and their contribution to normal cellular functions is more frequent than ever anticipated. 

An epigenetic modification is a dynamic process and has been proven to play a very important role in the development of cancer cells [45,46]. More interestingly, the identification of tumor-specific DNA methylation patterns in the plasma of patients has led to great efforts towards the non-invasive diagnosis of cancer [47,48]. These developments in the field of cancer investigation have provided additional convincing support that epigenetic differences may be present between the fetal DNA and the maternal DNA in maternal circulation during pregnancy.

## 3. DNA Methylation Biomarkers Discovery

The aim of DNA methylation-based approaches was first to identify fetal-specific methylation markers that would allow the discrimination of fetal DNA from the maternal DNA in maternal circulation and that have the potential to be developed into non-invasive prenatal diagnostic markers. The approaches that have been used for investigating the DNA methylation patterns in fetal DNA and maternal DNA are of three main categories: sodium bisulfite-based approaches, restriction enzyme-based approaches and methylated DNA immunoprecipitation-based approaches.

### 3.1. Sodium Bisulfite-Based Approaches

Sodium bisulfite conversion leads to the transformation of an epigenetic modification into a genetic sequence change for further investigation. More specifically, the treatment of DNA with sodium bisulfite results in the conversion of unmethylated cytosines to uracils, leaving methylated cytosines unchanged [49]. The genetic composition of the converted sequences of interest could be investigated using methylation-specific PCR (MSP) in which the amplification process is separate for the methylated (non-converted) fragments and the non-methylated (converted) fragments [50]. Alternatively, the methylation status of bisulfite converted sequences could be assessed through the implementation of sequencing technologies [49,51]. In 2002, Poon and his colleagues demonstrated for the first time the potential for the presence of epigenetic differences between the fetus and the mother by performing sodium bisulfite conversion of placental DNA and female peripheral blood DNA followed by MSP [50,52]. The first differentially methylated region was identified in 2005 by the use of sodium bisulfite conversion in combination with MSP and sequencing. The differentially methylated gene, known as *SERPINB5*, was found to be hypomethylated in fetal DNA and hypermethylated in maternal DNA [12]. The identification of hypomethylated fetal-specific *SERPINB5* sequences was also achieved in maternal plasma during pregnancy. This genomic region was used to demonstrate that fetal DNA is not detectable in maternal plasma 24 h after delivery [28].

Since then, great efforts have taken place from independent groups towards the identification of fetal-specific methylation markers. The initial attempts were based on the investigation of promoter regions and CpG islands. In 2008, a bisulfite based systematic search for placental DNA methylation markers on chromosome 21 was described. In this study, the methylation-sensitive single nucleotide extension (Ms-SNuPE) method was used to assess the methylation differences of CpG sites [53,54]. The above study performed an evaluation of the methylation status of 114 CpG islands (based on bioinformatics criteria) in five first trimester placental tissues and two samples of non-pregnant female blood. Among them, 22 CpG islands were identified as having the potential to be developed into biomarkers for the NIPT of trisomy [54].

In 2010, a second study was performed with the aim of identifying a panel of fetal-specific hypermethylated markers on chromosome 21, and it used the methylation pattern of a previously characterized gene, *RASSF1A*. The *RASSF1A* gene is located on chromosome 3 and has been found to be completely methylated in fetal DNA and completely unmethylated in maternal DNA. This characteristic allowed the use of the *RASSF1A* gene as a fetal universal marker [28,55]. The study was performed using the combined bisulfite restriction analysis (COBRA) [56] to investigate 35 gene promoter regions on chromosome 21. The analysis demonstrated that the *HLCS* gene located on chromosome 21 is fully methylated in placenta and unmethylated in maternal blood cells [15].

A recent report published in 2013 illustrates the potential of retrieving the methylation profiles of placental tissues and maternal blood cells using sodium bisulfite in combination with next generation sequencing technologies [10]. The investigators were able to retrieve the fetal methylome through the identification of single nucleotide polymorphism (SNP) genotype differences between the mother and the fetus in maternal plasma and to identify differentially methylated regions (DMRs). They identified 44,455 loci as being fetal-specific hypomethylated and 3081 regions as being fetal-specific hypermethylated. The above findings are in agreement with previous studies in which it was clearly evident that the fetal genome is mostly hypomethylated in contrast to the adult peripheral blood, which is greatly hypermethylated, indicating a regulatory role of the methylation patterns and gene expression profiles [44,57,58]. Interestingly, it has also been reported that hypomethylated sequences tend to be of a smaller fragment size. These findings could indicate a contribution of the fetal methylation status to the small fetal DNA fragments size in maternal plasma [10].

### 3.2. Restriction Enzyme-Based Approaches

Methylation patterns of CG dinucleotides can also be assessed using restriction enzymes, which have recognition sites containing CG sequences. Methylation-sensitive restriction enzymes can digest their recognition site only when unmethylated, whereas methylation insensitive restriction enzymes digest their recognition sites only when the cytokines of the CGs within their recognition site are methylated. In 2007, the team headed by Old reported for the first time the investigation and identification of a panel of differentially methylated regions on chromosome 21 using methylation-sensitive enzymes [59]. More specifically, the team used the HpaII enzyme, and the underlying idea was based on the fact that the enzyme would digest only the unmethylated type of its recognition site (CCGG). Therefore, this would allow them to identify regions containing the above recognition sites, which are differentially methylated between placenta and maternal blood cells. The study was focused on the investigation of promoters from highly expressed genes, randomly selected promoters, as well as randomly selected non-promoter regions. Among the 200 pre-selected regions, three promoter regions of the *AIRE*, *SIM2* and *ERG* genes were found to be methylated in the placenta and unmethylated in the maternal blood cells. The methylation status of those regions was confirmed by sodium bisulfite followed by MSP [59]. 

In 2011 a study performed by Peters and his team demonstrated that the use of methylation-based restriction enzymes, such as HpaII and MSpI, in combination with high-resolution arrays can distinguish differentially methylated regions between the placenta and maternal blood cells [58]. They presented a large panel of DMRs consisting of 6311 DMRs across chromosomes 13, 18 and 21 [58,60] and demonstrated that the fetal DNA is mostly hypomethylated, whereas the maternal blood cells are mostly hypermethylated, findings which are in agreement with previous reports [44,57]. Moreover, they illustrated that the majority of the hypomethylated regions of both fetal and maternal origin are located within CpG islands, promoters and exons, indicating a potential correlation with expression profiles [58].

### 3.3. Methylated DNA Immunoprecipitation-Based Approaches

One of the most modern methods of studying the levels of DNA methylation is the MeDIP (methylated DNA immunoprecipitation) approach. The method was first described in 2005 by Weber *et al*. with the aim of investigating the methylation pattern of cancer cells in a genome-wide fashion using microarray platforms [45]. In 2007, Beck and his team introduced linker-mediated PCR amplification (LM-PCR) in combination with the MeDIP methodology. They obtained large amounts of immunoprecipitated DNA and generated the first whole genome mammalian methylome using a large panel of different tissues [44,61]. The principles of the MeDIP methodology includes fragmentation of the DNA (through sonication or enzymatic digestion) into short DNA fragments of 300–1000 bp. The sample is denatured and incubated with a monoclonal antibody, which recognizes and attaches to the 5-methylcytosines of CpG dinucleotides. Immunoprecipitation of methylated sequences is accomplished with the addition of magnetic beads. Through the implementation of the MeDIP methodology, you can achieve direct enrichment of methylated fragments. Enrichment of methylated target sequences is easily retrieved through the use of a large number of different technologies, such as PCR, qPCR (quantitative Polymerase Chain Reaction), microarray and sequencing. Since its development, MeDIP has been extensively used for the investigation of the methylation status/patterns of cancer tissues with great success either in combination with microarray technologies (MeDIP-chip) [42,44,45] or, more recently, in conjunction with next generation sequencing (MeDIP-seq) [62,63,64,65].

The MeDIP methodology was first introduced to the field of NIPT by our team in 2009 with the aim of investigating and identifying DMRs between placenta and female peripheral blood towards the development of NIPT for the identification of common aneuploidies [57]. Our team used MeDIP in combination with chromosome-specific high-resolution oligo arrays for the investigation of the methylation pattern of chromosomes 13, 18, 21, X and Y. Although previous studies solely investigated promoter regions and CpG islands for DMR identification, we were the first to screen entire chromosomes of interest irrespective of the genomic position or CG content. At the time, we reported the largest panel of DMRs with the potential to be developed into NIPT biomarkers for the most common fetal aneuploidies. More specifically, we identified around 2000 DMRs on each of the chromosomes investigated, and interestingly, we noticed that the vast majority of the DMRs were located within non-genic regions and in relatively poor CG regions. More specifically, we illustrated that 56%–83% of the DMRs were located within non-genic regions, whereas only 1%–11% were located within CpG islands. Our findings were concordant with previous studies performed by other groups investigating a panel of different tissues [44] and were also in agreement with more recent reports using bisulfite sequencing technologies [3,58]. We were also able to report the presence of inter-individual variability and the changes in the methylation patterns during the progression of the pregnancy, findings which have recently been confirmed by independent groups [10]. 

Following our study, the group headed by Chim used MeDIP in combination with a microarray platform targeting promoter regions and CpG islands. The group identified a panel of eight DMRs with the potential of being developed into biomarkers for diagnostic purposes [66], most of which are among the DMRs identified previously by our group [57]. Any discrepancies reported regarding the identification of DMRs, such as the failure to have the exact same methylation status of all DMRs reported by independent studies, are not uncommon, since different platforms and different methylation-based technologies were used.

## 4. Implementation of Methyl-Biomarkers in NIPT

The discovery of DMRs has mainly been focused on chromosomes 13, 18, 21, X and Y with the aim of identifying as a priority methylation-based biomarkers (methyl-biomarkers) suitable for the development of NIPT for the most common chromosomal fetal aneuploidies. The first attempt was reported back in 2006 for the NIPT of trisomy 18 (Edward’s syndrome) [67]. In this study, the authors implemented a combination of sodium bisulfite conversion with MSP using maternal plasma samples from normal and trisomy 18 pregnancies. To achieve discrimination, they used the information of an SNP located within the *SERPINB5* gene. The cases were considered informative if the SNP was homozygous in the mother and heterozygous in the fetus, and only those cases could be used for NIPT of trisomy 18 (T18). To achieve this, the team introduced the so-called epigenetic allelic ratio (EAR) in which the chromosome 18 copy number was assessed based on the allele ratio calculation of an informative SNP. The challenge in this study was to have informative SNPs, and because there was only a single SNP in the target sequence, it was extremely difficult to be informative in all cases tested (Table 1). The results showed that among the 173 euploid placentas and 14 trisomy18 placentas genotyped for the polymorphism, only 31 and seven placentas, respectively, were informative. The rarity of having an informative SNP in this study does not allow this approach to be implemented population-wide [23,26].

To overcome the above limitations, in 2010, the same group developed an SNP-free methylation-based assay for NIPT of trisomy 21 (Down syndrome). Methylation-sensitive restriction digestion was used followed by digital PCR to investigate DMRs identified on chromosome 21 [15]. The copy number of chromosome 21 was determined through the epigenetic-genetic (EGG) chromosome dosage approach using the fetal-specific hypermethylated promoter region of the *HLCS* gene located on chromosome 21 and the *ZFY* locus on chromosome Y. The assay tested 24 maternal plasma samples from euploid pregnancies and five maternal plasma samples from trisomy 21 pregnancies. All but one euploid pregnancy were correctly classified (Table 1) [15]. 

The EGG chromosome dosage approach was also implemented for the NIPT of trisomy 18 in which the fetal-specific methylated *VAPA-APCDD1* loci on chromosome 18 and the *ZFY* on chromosome Y were quantified with digital PCR after HinP1I- and HpaII sample digestion [66]. The study was performed on nine maternal plasma samples from male trisomy 18 pregnancies and 27 maternal plasma samples from male euploid pregnancies. Among them, eight out of nine and one out of 27 trisomy 18 and euploid pregnancies, respectively, were correctly identified, which corresponds to 88.9% sensitivity and 96.3% specificity (Table 1) [66]. 

**Table 1:** Comparison of different methylation-based approaches towards the non-invasive prenatal tests (NIPT) of aneuploidies. EAR, epigenetic allelic ratio (EAR); EGG, epigenetic-genetic; SNP, single nucleotide polymorphism.

Assay	Technology	Sample size	Sensitivity/Specificity (%)	Advantages	Disadvantages	Reproduced by others
EAR on chromosome 18 [67]	Sodium bisulfite, digital PCR	2 normal 2 trisomy 18	Not defined/not applicable population-wide	Applicable irrespective of gender	Requires informative SNP, depends on the bisulfite conversion performance	No
EGG on chromosome 21 using *ZFY* [15]	* COBRA, digital PCR	24 normal 5 trisomy 21	95.8% specificity 100% sensitivity	SNP-free assay	Applicable only to male pregnancies, depends on the digestion and bisulfite conversion efficiency	No
EGG on chromosome 18 using *ZFY* [66]	* COBRA, digital PCR	27 normal 9 Trisomy 18	96.3% specificity 88.9% sensitivity	SNP-free assay	Applicable only to male pregnancies, depends on the digestion and bisulfite conversion efficiency	No
EGG on chromosome 21 using *TMED8* [68]	** MRED digestion, digital PCR	33 normal 14 trisomy 21	Variable depending on the fetal allele	Applicable irrespective of gender	Requires informative SNP, applicable only to male pregnancies, depends on the digestion efficiency	No
Fetal-specific DNA methylation ratio on chromosome 21 (1st study) [69]	*** MeDIP, real-time qPCR	40 normal 40 trisomy 21	100% specificity 100% sensitivity	Applicable irrespective of gender and SNPs	Depends on MeDIP performance	Yes [70,71]
Fetal-specific DNA methylation ratio on chromosome 21 (2nd study) [72]	*** MeDIP, real-time qPCR	125 normal 50 trisomy 21	99.2% specificity 100% sensitivity	Applicable irrespective of gender and SNPs	Depends on MeDIP performance	No
Bisulfite sequencing [10]	Sodium bisulfite, next generation sequencing	7 normal 5 trisomy 21	100% specificity 100% sensitivity	Applicable irrespective of gender and SNPs	Depends on bisulfite conversion efficiency	No

* Combined bisulfite restriction analysis; ** methylation restriction enzymatic digestion; *** methylated DNA immunoprecipitation.

Although the results from the studies using the EGG chromosome dosage approach were promising, the technology was restricted to male pregnancies, because the EGG calculation involved the use of the *ZFY* gene (Table 1). To overcome the above difficulties, a modification was introduced in the EGG calculation to be able to include the testing of female pregnancies, as well. The study was performed using 14 maternal plasma from trisomy 21 pregnancies and were compared to 33 cases with a euploid fetus [68]. For calculation purposes, the *ZFY* gene was replaced with an autosomal genetic reference marker. Interpretation of the results was achieved using a paternally-inherited SNP allele on the *TMED8* gene located on chromosome 14, which served as a baseline for the EGG chromosome dosage calculation. The sensitivity of the assay varied depending on which of the two alleles of an SNP was fetal-specific, making the evaluation of the assay performance even more challenging. Overall, although the limitation of testing only male pregnancies was overcome, the assessment of the copy number of chromosome 21 remained a challenge, as the presence of at least one informative SNP was necessary (Table 1).

A different approach was proposed by our group in 2011 and was based on using the MeDIP methodology in combination with real-time quantitative PCR (real time-qPCR) for the quantification of selected DMRs located on chromosome 21 [69]. We selected 12 previously identified DMRs located on chromosome 21 [57], which were hypermethylated in fetal DNA and hypomethylated in female peripheral blood cells. We used in our study a total of 40 maternal blood samples from euploid pregnancies and 40 maternal blood samples from trisomy 21 cases. We developed a diagnostic formula by calculating the DNA methylation ratio of the selected DMRs using 20 normal pregnancies and 20 trisomy 21 pregnancies. Eight specific DMRs were the most statistically significant markers in discriminating normal from trisomy 21 pregnancies. The MeDIP-qPCR methodology was used to then test 40 additional pregnancies, of which 20 were obtained from trisomy 21 pregnancies and showed 100% specificity and 100% sensitivity [69]. We also demonstrated that diagnostic accuracy can only be achieved through the combination of multiple DMRs from chromosome 21, which was an important finding for further NIPT developments [23]. 

Our team continued to improve the MeDIP-qPCR assay with a larger validation study of 175 pregnancies that included 50 trisomy 21 pregnancies [72]. In this larger-scale validation, we re-evaluated our diagnostic assay, taking into consideration the genomic composition of our DMRs and by selectively excluding those DMRs located in copy number variable (CNV) regions. Based on the above, we re-designed our diagnostic formula and then evaluated its performance using 100 new cases, which included 25 trisomy 21 pregnancies. The results demonstrated 100% sensitivity and 99.2% specificity (Table 1) [72]. Our group also investigated whether the variability of the fetal fraction present in maternal plasma has a negative effect in our assay’s diagnostic efficiency. Although previous reports demonstrated an effect of different fetal amounts present in maternal plasma [73,74,75], our study has shown no significant association between cffDNA fraction, absolute fetal amount or the concentration present in maternal plasma with the test result classification using our diagnostic formula [20,72]. We speculate that this is due to the fact that maternal blood contains <1% of fetal DNA [20,72] in contrast to maternal plasma, which contains ~10%–15% fetal DNA [10,76].

More importantly, the results of our studies have been reproduced by two independent groups, which have reported their results using the MeDIP-qPCR methodology and the published diagnostic formula [70,71]. In addition, independent groups have also commented positively on the potential prospects or application of the MeDIP-qPCR assay towards the NIPT of chromosomal aneuploidies. The low cost of the technology and the ease of implementing it, in combination with the use of equipment common to every laboratory, allows its implementation in any diagnostic laboratory setting [77]. A major strength of the MeDIP-qPCR assay is that it is a gender- and polymorphism-independent assay that could be implemented population-wide. Nevertheless, a different independent group has failed to reproduce the MeDIP-qPCR results by performing a small scale validation study [78]. Lack of reproducibility of the results would not be a surprise to our team, since, as stated in our reply to the above manuscript, very stringent quality control criteria must be applied to critical reagents and conditions throughout the method [79].

A very interesting recent development of investigating DNA methylation for use in NIPT has been the implementation of sodium bisulfite DNA treatment in combination with next generation sequencing technologies (NGS) [10]. The study is presented as a proof of principle and demonstrates one use of the assay with the detection of trisomy 21. NGS technologies have already been introduced in the field of NIPT by different independent groups with the primary aim of detecting the most common chromosomal aneuploidies [73,74,75,76,80,81,82]. Biotechnology companies have already introduced in the market their NGS-based NIPT of the most common chromosomal fetal aneuploidies [83,84,85]. However, sequencing of maternal plasma can turn out to be very challenging, due to the restrictions of the very low amount of fetal DNA available. Furthermore, such technology is not yet available in all clinical laboratories. Sequencing technologies are still considered to be of a high cost, requiring significant infrastructure, are labor intensive and require highly trained personnel, and the bioinformatics analysis can be very challenging, especially when the target sequence is of a very low amount, such as fetal DNA present in maternal plasma.

## 5. Evaluating the Efficiency of Methylation Assays

Developments towards methyl-biomarker discovery and their applications in the NIPT of fetal chromosomal abnormalities were achieved through a number of independent groups, as described above, using different methylation-based approaches. Different analytical tools and a variety of quantitative approaches (e.g., MSP, digital PCR, real-time qPCR, microarray platforms and next generation sequencing) were used, of which the statistical power in discriminating normal from abnormal pregnancies has been extensively assessed [23,26,86]. Nevertheless, the statistical discriminating power of each of the end point analytical tools relies on the efficiency of the methylation-based technology used to enrich the fetal DNA in maternal circulation (Table 1). Therefore, the evaluation and assessment of the efficiency of the methylation-based enrichment technology used is of significant importance. 

One of the most commonly used approaches is the treatment of DNA with sodium bisulfite. Sodium bisulfite conversion is considered the gold standard in the evaluation of the methylation status of different tissues and has been extensively used, especially in the field of cancer [87,88]. However, it is well known that this chemical treatment of the DNA is associated with a high degree of DNA degradation, reaching >90% of the template DNA [89]. This major drawback of the technology is undesirable for its implementation in plasma samples of pregnant women. During pregnancy, the amount of fetal DNA in maternal plasma is very low [10,76], and further degradation will result in even fewer fetal DNA molecules available for quantification; therefore, the accuracy and sensitivity of the test will be reduced. To compensate for the degradation effect, much larger amounts of maternal plasma are required, which makes the testing of maternal plasma even more complicated. Furthermore, bisulfite conversion can be challenging, since 100% conversion of the unmethylated cytosines to uracils is rarely achieved, and purification is required to remove the sodium bisulfite [90]. Such an effect will bias the correct interpretation of the results [23]. On the other hand, bisulfite conversion strategies are not sensitive to low purity and low integrity samples, an advantage especially for samples with very low starting DNA amounts. Nevertheless, bisulfite conversion in combination with sequencing technologies can provide a comprehensive analysis of the methylation status at the base pair composition, which can make it a very powerful tool (Table 2).

**Table 2:** Comparison of different methylation assays.

Methylation assay	Advantages	Disadvantages	Analytical tool used for NIPT
Sodium bisulfite	Not sensitive to sample impurities, methylation analysis at the base pair level	DNA degradation (>90%), 100% conversion is rarely achieved	* MSP, microarrays, Digital PCR, ** COBRA, *** NGS
Restriction enzyme digestion	Easy to perform and low cost	Sensitive to sample impurities, requires high amount of starting DNA, applicable to a limited number of DNA sequences	** COBRA, digital PCR
**** MeDIP	Ideal for investigating low CG content regions, low cost assay, not sensitive to sample impurities, can be applied with low starting DNA amounts	Depends on antibody efficiency and ideal combination of affinity reagents	Real time-qPCR, microarrays

* Methylation-specific PCR; ** combined bisulfite restriction analysis; *** next generation sequencing; **** methylated DNA immunoprecipitation.

A different approach implemented by a number of independent groups towards methyl-biomarker discovery and methylation-based NIPT developments has been the use of methylation restriction enzymes, as described above. Through methylation restriction enzymatic digestions (MRED), the unmethylated maternal origin sequences, present in maternal plasma, are digested to achieve indirect enrichment for the corresponding sequences of fetal origin, which are methylated. The efficiency of the MRED assays depends on the purity of the sample, and for this reason, they require high purity and high integrity samples [90]. Additionally, MRED assays require fairly high quantities of starting material, which is a restriction to its implementation in plasma samples, because not only the target fetal DNA sequences are of a low amount, but also the total plasma DNA is very low (around 10 ng/4 mL plasma) [20]. An additional drawback of the assay is that it can only evaluate the methylation status of a specific and very limited number of genomic sequences. Only those sequences that include a recognition site of a methylation-dependent restriction enzyme could be evaluated. Such inherent restrictions do not allow efficient and detailed genome-wide methylation assessment [23,26]. An example is the recognition sites of the HpaII restriction enzyme, which are presented in only 3.9% of CGs across non-repetitive sequences of the human genome [91]. Moreover, the efficiency of digestion should always be carefully evaluated for an unbiased interpretation of the results. Nevertheless, it is a very easy to perform assay and low cost.

The MeDIP assay, an affinity-enrichment method, was also utilized towards DMR identification and characterization to discriminate fetal DNA from maternal DNA in maternal circulation during pregnancy. Based on studies performed by several independent groups, it is clearly evident that the vast majority of DMRs identified between different tissues are located within non-genic and CG poor regions [44,58]. Based on recent reports, the MeDIP methodology is ideal for the investigation of low CpG density regions [92]. Indeed, the DMRs identified and selected for NIPT of trisomy 21 using MeDIP-qPCR are located in low CpG sites and are mostly found within non-genic regions [57,69,72]. Therefore, we strongly feel that MeDIP is the choice of selection for the investigation of DMRs towards NIPT. MeDIP is an efficient method for genome-wide methylation assessment [42,44,45], as it can evaluate the methylation levels irrespective of genomic composition and overcomes limitations of the previously described methodologies. The MeDIP assay can tolerate sample impurities, and thus, no prior sample purification is required. Furthermore, it has recently been proven to be applicable for low starting DNA templates, generating sufficiently enriched outputs [64,65], a development that simplifies and makes possible its implementation with plasma samples. Moreover, it is a technically robust methodology, easy to use and affordable. Nevertheless, the efficiency and performance of MeDIP greatly depends on determining the ideal combination of affinity reagents. This is very important, especially in regions with varying methylcytosine density, such as the DMRs identified for the NIPT of common chromosomal aneuploidies [57,69,72]. The advantages and disadvantages of all the different methylation-based assays implemented towards the NIPT of fetal chromosomal abnormalities are summarized in Table 2.

## 6. Conclusions and Future Directions

Deciphering the epigenome and understanding the underlying mechanisms that lead to epigenetic modifications has been one of the most interesting fields under investigation for the last decade. Since 2002, a large panel of DMRs has been identified by independent groups, with the potential of being developed into diagnostic markers having as a primary goal the development of NIPT for common fetal chromosomal abnormalities. 

We speculate that epigenetic approaches towards NIPT will soon dominate the field of NIPT, because they are easy to perform, are fast and inexpensive compared to existing NIPT approaches, which are based on next generation sequencing technologies [73,74,75,81,82]. We speculate that one of the first epigenetic-based approaches that will be launched for the NIPT of common chromosomal fetal abnormalities will be a MeDIP-based approach. NIPD Genetics Ltd., a company in which three of the authors are employed, is dedicated to developing a MeDIP-qPCR-based diagnostic assay. The company will be soon ready to launch the first epigenetic-based NIPT for trisomy 21 following completion of a large-scale validation study [23,72,93].

Methylation-based approaches could also be used for retrieving the methylation status of abnormal tissues, such as placental tissues from aneuploid pregnancies. A very recent study has shown that trisomy 21 placentas are characterized by a global hypermethylation in contrast to normal placentas, which are mainly hypomethylated [94]. Identifying such disease-associated characteristics can benefit and contribute to more robust and sensitive NIPT. Furthermore, methylation differences during fetal development have also been shown to be associated with transcription. It has been demonstrated that the early gestational placental methylome is significantly associated with gene expression [58]. Such structural and regulatory characteristics of the placental epigenome are of great importance and could be used to determine the role of aberrant or altered methylation in placental dysfunction.

In addition to the methods described in this review, the implementation of alternative methylation-based approaches, such as MBD (methylated binding domain) [92] and McrBC (a GTP-requiring, modification-dependent endonuclease of *Escherichia coli* K-12) fragmentation, as well as HELP (HpaII tiny fragment enrichment by ligation-mediated PCR) [95,96], in combination with the development of bioinformatics-based algorithms, will contribute to a better understanding of the fetal methylome. We envision that epigenetic-based enrichment methods will have a major contribution to fetal methylome analysis through direct testing of maternal plasma. Looking ahead, we predict that epigenetic-based approaches in combination with genetic-based approaches and advanced technological approaches, such as digital PCR and next generation sequencing, will contribute to the development of NIPT of more subtle fetal abnormalities, such as point mutations, microdeletion/microduplication syndromes, *etc*.
